# Supplementary material for: Complexome profiling of the Chlamydomonas psb28 mutant reveals TEF5 as an early PSII assembly factor
Source: Plant Cell. 2025 Mar 17;37(6):koaf055. doi: 10.1093/plcell/koaf055 (PMC12164590; doi:10.1093/plcell/koaf055)
Supplement: koaf055_Supplementary_Data [file koaf055_supplementary_data.zip › psb28_interactive.zip!/plots/intensity/qualityPlots/peptides/CPN20_Cre08.g358562.t1.1.html]

### Cre08.g358562.t1.1

Trivial name: CPN20  
SeqCoverage [median]: 30.10  
Mass: 22.77 kDa  
  
 

| Sequence | MissedCleavage | Mass [Da] | wtIntensity | muIntensity | Score |
| --- | --- | --- | --- | --- | --- |
| IAQLKPLSDR | 1 | 1139.67 | 1889.22 | 9925.74 | 74.94 |
| NKPTAGSIIALGDAK | 1 | 1454.81 | 2269.30 | 10011.07 | 107.69 |
| SVGGVLLPASVR | 0 | 1153.68 | 1936.20 | 16068.24 | 121.90 |
| VLVKVDKEEAK | 2 | 1256.73 | 0.00 | 1274.49 | 74.94 |
| YSGTEFEEDGDNYIVVR | 0 | 1991.87 | 0.00 | 731.80 | 151.10 |
|  |  |  | 6094.7 | 38011.3 |  |
